# Potentials of straw return and potassium supply on maize (*Zea mays* L.) photosynthesis, dry matter accumulation and yield

**DOI:** 10.1038/s41598-021-04508-w

**Published:** 2022-01-17

**Authors:** Ya-fang Fan, Ju-lin Gao, Ji-ying Sun, Jian Liu, Zhi-jun Su, Shu-ping Hu, Zhi-gang Wang, Xiao-fang Yu

**Affiliations:** 1 College of Agronomy, Inner Mongolia Agricultural University, No.275, XinJian East Street, Hohhot, 010019 China; 2 Vocational and Technical College, Inner Mongolia Agricultural University, Baotou, 014109 China

**Keywords:** Plant sciences, Ecology

## Abstract

Maize (*Zea mays* L.) is considered one of the most important grains in the world. Straw return has the effect of reducing soil bulk density and increasing soil porosity. Straw returning and potassium fertilizer can supplement soil potassium content. The improvement of soil structure and the optimization of soil nutrient levels provide a good environment for high yield and high efficiency of maize. Therefore, three field experiments were carried out over a three-year period (2018–2020) to study the effects of straw returning on photosynthesis, dry matter accumulation and yield of maize 'Xianyu 335' under two different fertilization methods and four potassium application levels. The results showed that straw returning and potassium application had significant effects on the above indicators. The above indicators were significantly improved by deep tillage straw returning compared with no tillage straw returning. Increasing potassium supply can promote the effect of straw returning. The photosynthesis, dry matter accumulation and yield parameters of maize treated with straw returning and deep tillage combined with 60 kg/hm^2^ potassium fertilizer (SFK60) reached the highest in the three harvest seasons. The corn planting profit of SFK45 treatment is the highest, which is $1868.92 per ha. Therefore, SFK45 is an effective way to ensure stable and high yield of corn and maximize farmers' income.

## Introduction

Maize is consumed as an important strategic material with multiple significant values such as grain, economy, feed, and energy^1^. The maize yield is regulated not only by its photosynthesis and dry matter accumulation^2,3^, but also by external cultivation measures and fertilizer supply^4–6^. Straw return is a green and sustainable agricultural cultivation technology. China produced the most crop residue in the world, approximately 8.4 × 10^8^ t^7^. Straw contains a large amount of nitrogen, phosphorus, potassium and other nutrients^8^. Straw return could take plant potassium to soil, so that replenish soil potassium effectively and improve soil nutrient status^9^. Straw return could improve soil properties soil structure^10^. Yan et al.^11^ showed that straw return has the effect of reducing soil bulk density and increasing soil porosity through a 5-year positioning test. The optimization of soil nutrient level and the improvement of soil structure provide a good environment for high yield and high efficiency of maize. Rational use of straw resources is an important way to realize the sustainable development of agriculture^12^. Straw mulching with no tillage considers a traditional conservation measure for improving crop microclimate^13,14^. Continuous straw mulching with no tillage is not conducive to changing soil plow bottom and even causes a poor sowing quality^15,16^. Straw return with deep tillage is one of the most important agricultural management measures to break soil plow bottom and improve sowing quality to increase maize photosynthesis and yield^17^. Fertilizer also plays an important role in the process of achieving stable and high maize yield, just like cultivation measures. Potassium fertilizer can maintain the suitable state of photosynthetic indexes such as Pn, Gs, Tr and Ci of spring maize, and maintain a longer high photosynthetic duration, effectively improve the photosynthesis attributes of maize, promote dry matter accumulation, and achieve stable and high yield of maize^18–20^. Potassium fertilizer made a pivotal contribution to ensure the steady increase of maize yield^21,22^. Different potassium supplies influenced the maize yield variously. Studies have shown that the potassium supply of high-yield maize was concentrated in the range of 40–80 kg/ha^23^. Yan^24^ reported that the optimum potassium supply for high maize yield in medium-fertility soil was 39.5 kg/ha. Straw mulch and potassium application contributed to the increase of maize yield. However, the decomposition rate of straw was slow during straw mulching, so the effect of straw mulching combined with potassium fertilizer was not as good as that of straw deep plowing combined with potassium fertilizer^25^. Zhao^26^ considered that the interaction between straw return and potassium fertilizer was the best potassium fertilizer management mode to achieve a high yield of maize. Therefore, standardizing straw returning methods and potassium supply will become a significant measure for high maize yield.

To sum up, previous studies mainly focused on the effects of straw returning on maize photosynthesis and yield, and the effects of potassium fertilizer on maize yield. However, there was a lack of research on the cooperation of different straw return methods and potassium fertilization levels. Therefore, the basis on previous studies, we conducted the field experiments for three years to determine the effects of straw return and potassium supply on maize photosynthesis, dry matter, and yield in the Tumochuan Plain irrigation area in Midwestern Inner Mongolia of China. The main objective of this study was to understand how different straw return methods and potassium fertilization levels could influence maize growth and yield. Specifically, we tested (1) how different straw return methods and potassium fertilization levels influenced maize photosynthesis, dry matter, and yield? (2) What was the best treatment for obtaining a high yield? (3) What was the best method for maximizing the income of farmers? (4) Whether the effects of the treatments varied among the three years? The information generated in this study will be helpful to select the best agricultural measure that can maintain a high yield and obtain the maximum profit.

## Materials and methods

### Site description

Three field experiments were carried out at the experimental base of Inner Mongolia Agricultural University (40°33′ N, 110°31′ E) located in Inner Mongolia during the seasons from 2018 to 2020. The three-year experiments were carried out in the same plot, and the test of straw return and potash fertilizer started in 2016. The surface soil fertility (0–20 cm) and the climatic conditions during the growth period of maize were shown in Table 1.

**Table 1:** Soil fertility and climatic conditions.

Year	Organic matter (g/kg)	Total N (g/kg)	Available N (mg/kg)	Available *P* (mg/kg)	Available K (mg/kg)	pH	Sunshine hour (h)	Average temperature (℃)	Average rainfall (mm)
2018	25.53	1.1	92.35	9.8	117.37	7.6	1869.2	20.9	215.2
2019	25.96	1.1	93.65	9.7	120.95	7.6	1893.6	20.7	213.5
2020	26.29	1.1	91.28	10.2	124.53	7.6	1825.7	21.2	235.7

The experimental research and field studies on plants (either cultivated or wild), including the collection of plant material, are comply with relevant institutional, national, and international guidelines and legislation. The field study was carried out on the official land which belonged to the key laboratory of crop cultivation and genetic improvement of Inner Mongolia Autonomous Region, permission was given after the research application passing verification, studies were comply with local and national regulations. During the field study, our test did not involve endangered or protected species. No specific permissions were required for conducting the field study because it was not carried out in a protected area.

### Experimental design

The test material was maize ‘Xianyu 335’. A split-plot design with five replications was used. The two straw return methods were assigned in the main plots, which were straw return with deep tillage (SF) and straw mulching with no tillage (FG). The four potassium fertilization levels (0, 30, 45 and 60 kg/ha) were allocated in the sub-plots, which were expressed by K0, K30, K45 and K60. CK was the control treatment which straw was not returned and without potash supply. The treatments of this experiment were as follows: SFK0, SFK30, SFK45, SFK60, FGK0, FGK30, FGK45 and FGK60. Each sub-plot consisted of 10 rows with 5 m length and 0.6 m width, and each sub-plot area was 30 m^2^ in the three years. The designated tillage practices were performed each autumn after the harvest of maize ‘Xianyu 335’. Straw return treatment used the straw secondary crushing technology. Firstly, John Deere W210 combine harvester was used to harvest and crush maize straw for the first time in autumn, then 4Q-1.5-type Straw Stalk Grinder was used to crush maize straw for the second time. The maize straws were mechanically chopped into 3–6 cm long pieces. The treatments of straw return with deep tillage returned maize straw pieces into the field with a depth of 40 cm, raked and compacted. The above operations were to prevent the straws from being too long and the soil pressure was not solid, which could affect the emergence and growth of spring maize. The treatments of straw mulching with no tillage covered the topsoil with maize straw pieces. The straw return methods were mainly carried out after harvest, which created good conditions for high-quality sowing in spring and maize growth and development.

The maize was seeded on April 25, 26 and 24, and was harvested on October 3, 5 and 2 in 2018, 2019 and 2020, respectively. Base fertilizer applied at the seeding included P_2_O_5_ at the rate of 105 kg/ha, and K_2_O of different application levels of 0, 30, 45 and 60 kg/ha. In addition, 300 kg/ha of N was top-dressed during the jointing stage. A series of cultivation and management measures such as irrigation and weeding was carried out according to the local high-yield cultivation^27^. Economic analysis of results was used to determine the variances between different factors to obtain the greatest profitability of straw return methods and potassium supply. The profit of maize planting was calculated according to the local market price of maize (1yaun/kg) and the harvest time of the production. The production costs included the expenses of different straw return methods, potassium supply, seeds, hoeing and watering, which were calculated at the local market price. The economic analysis was evaluated based on gross income, net income and benefit cost ratio (Table 2).

**Table 2:** Itemization of maize planting cost of different treatments during 2018 to 2020.

Treatment	Cost (yuan/ha)					Gross income (yuan/ha)	Net income (yuan/ha)	Benefit cost ratio (%)			
	Straw return	Potash fertilizer	Seeds	Hoeing and watering	Total cost			Straw return	Potash fertilizer	Seeds	Hoeing and watering
CK	0	0	950	750	1700	11,773.95	11,773.95	0.00	0.00	8.78	6.80
SFK0	750	0	950	750	2450	12,945.21	12,195.21	6.15	0.00	7.92	6.15
SFK30	750	120	950	750	2570	13,693.02	12,943.02	5.79	0.88	7.46	5.79
SFK45	750	180	950	750	2630	14,718.77	13,968.77	5.37	1.24	6.90	5.37
SFK60	750	240	950	750	2690	14,744.39	13,994.39	5.36	1.65	6.89	5.36
FGK0	450	0	950	750	2150	12,537.53	12,087.53	3.72	0.00	8.20	6.36
FGK30	450	120	950	750	2270	13,379.29	12,929.29	3.48	0.91	7.64	5.94
FGK45	450	180	950	750	2330	13,991.39	13,541.39	3.32	1.30	7.28	5.66
FGK60	450	240	950	750	2390	14,292.64	13,842.64	3.25	1.71	7.12	5.54

### Measurements

Photosynthetic parameters^28^. In the silking stage (R1), the photosynthetic parameters of ear leaves from five healthy and uniform plants in each plot were measured by using a portable photosynthesis system (LI-6400XT, USA) on sunny days. Before I start measuring, I adopted the open-air path and built-in light source, and the light intensity is set to 1500 μ mol CO_2_ m^2^ s^-1^. Then I used a leaf in the leaf chamber of the instrument and pressed the "start measurement" button on the operation panel. The net photosynthetic rate (Pn), stomatal conductance (Gs), transpiration rate (Tr), intercellular CO_2_ concentration (Ci) of ear leaves could be measured within about one minute. Click the save button when the measurement is completed, and then proceed to the measurement of the next cell.

Dry matter accumulation^29,30^. Maize plants were taken in each plot during R1 and R6 stage with five replicates. Maize plants were dried at 105 ℃ for 30 min, then dried at 80 ℃ to constant weight, and weighed the dry matter weight.

Yield and yield component^31,32^. At the physiological maturity stage (R6), four rows in the middle of the measured production area were selected, and all plants in these rows were harvested after the removal of the side plants. Ten plants with uniform ear growth were selected for determination of ear rows, row grains, 1000-grain weight, and grain water content (measured with an LDS-1G moisture content detector), then calculated the maize yield.

### Statistics analysis

Data SPSS window version 17 (SPSS Inc., Chicago, USA) was used to finishing statistical analysis and correlation analysis. Under straw return methods, potassium fertilization levels, and test years, we examined photosynthetic characteristics, dry matter accumulation and yield using GLM based on the model for a split-plot design^33,34^. The values were all the F-values of the ANOVA. Straw return methods, potassium fertilization levels, and test years were the independent variables, and the photosynthetic characteristics, dry matter accumulation and yield were dependent variables in this test. In order to determine the impact of independent variables on dependent variables, statistically significant variance was tested using two-way analysis of variance, and multiple comparisons were made using the least significant difference (LSD) test with α = 0.05^35^. Histograms were conducted by using Sigma Plot 12.5. And different letters on histograms indicated that means were statistically different at *P* < 0.05 level.

## Results

### Significance tests of straw return methods, potassium fertilization levels and their interactions

Analysis of variance (ANOVA) results showed that straw return methods and potassium fertilization levels had significant effects on maize photosynthesis, dry matter and yield from 2018 to 2020 (Table 3). Significant interactions between straw return methods and potassium fertilization levels were only found on Pn of 2018 and 2020, and Tr of 2018–2020. Through the comparison of three-year F-values, it could be found that the effect of potassium fertilization levels on maize photosynthesis, dry matter and yield was greater than that of straw return methods.

**Table 3:** Significance of the effects of straw return methods, potassium fertilization levels and their interactions on maize growth and yield using ANOVA.

Year	Source	Pn (μmol·m^-2^·s^-1^)	Gs (mmol·m^-2^·s^-1^)	Tr (mmol·m^-2^·s^-1^)	Ci (μmol·mol^-1^)	Dry matter in R1 (kg/ha)	Dry matter in R6 (kg/ha)	Yield (kg/ha)
2018	S	31.2**	24.28**	76.15**	6.6*	9.24**	11.01**	2.21 ns
	K	51.14**	67.78**	195.24**	10.74**	20.21**	34.07**	7.71**
	S × K	5.93**	1.09 ns	9.13**	0.34 ns	0.79 ns	0.24 ns	0.07 ns
2019	S	12.45**	27.71**	45.74**	4.66*	5.55*	9.46**	4.89*
	K	14.49**	114.66**	115.35**	14.14**	13.76**	28.22**	14.59**
	S × K	2.13 ns	0.53 ns	4.17*	0.32 ns	0.54 ns	0.22 ns	0.24 ns
2020	S	27.55**	22.23**	38.02**	6.91*	6.48*	13.93**	6.29*
	K	40.02**	92.37**	77.3**	22.09**	19.06**	45.56**	16.02**
	S × K	4.03*	0.17 ns	3.27*	0.32 ns	1.23 ns	0.89 ns	0.26 ns

Numbers were F-values. Stars indicated the level of significance (* = *p* < 0.05, ** = *p* < 0.01), ns represented insignificant. S represented straw return methods, including SF and FG; K represented potassium fertilization levels, including K0, K30, K45, K60 kg/ha.

### Effects of straw return and potassium fertilizer on photosynthesis of maize

The straw return methods and potassium fertilization levels significantly influenced (*p* ≤ 0.05) the maize photosynthesis compared to control (CK), resulting in Pn, Gs and Tr values that were higher than those of CK, and Ci value that was lower than that of CK.

**Figure 1:**
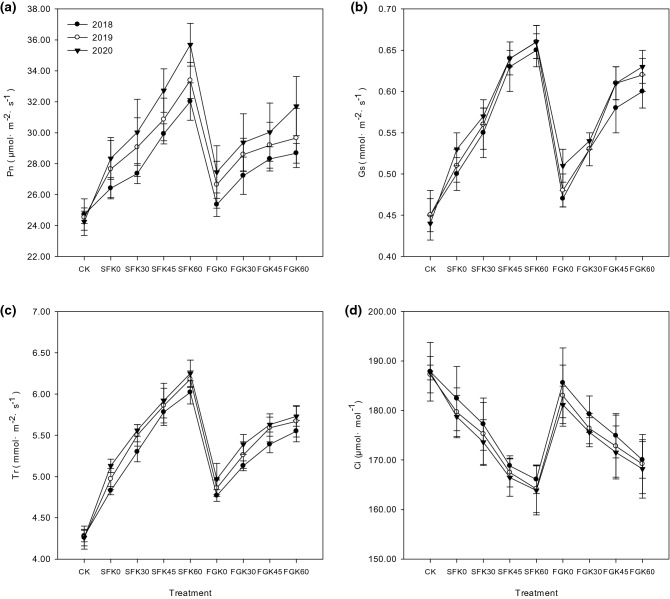
Effects of straw return methods and potassium fertilization levels on maize photosynthesis.

Straw return and potassium supply increased Pn, Gs and Tr. From 2018 to 2020, compared with CK, Pn increased by 1.70–4.09 under SFK0, 2.65–5.77 under SFK30, 5.21–8.48 under SFK45, 7.31–11.44 under SFK60, 0.63–3.20 under FGK0, 2.50–5.11 under FGK30, 3.60–5.79 under FGK45, and 3.97–7.47 μmol·m^-2^·s^-1^ under FGK60 (Fig. 1a). Gs increased by 0.60–0.90 under SFK0, 0.10–0.13 under SFK30, 0.18,-0.19 under SFK45, 0.20–0.22 under SFK60, 0.02–0.06 under FGK0, 0.08–0.09 under FGK30, 0.13–0.17 under FGK45, and 0.15–0.19 mmol·m^-2^·s^-1^ under FGK60 (Fig. 1b). Tr increased by 0.55–0.87 under SFK0, 1.02–1.30 under SFK30, 1.51–1.67 under SFK45, 1.74–1.99 under SFK60, 0.49–0.71 under FGK0, 0.86–1.13 under FGK30, 1.12–1.38 under FGK45, and 1.27–1.47 mmol·m^−2^·s^−1^ under FGK60 (Fig. 1c).

Straw return and potassium supply decreased Ci. From 2018 to 2020, compared with CK, Ci decreased by 5.43–8.92 under SFK0, 10.59–14.05 under SFK30, 19.04–21.21 under SFK45, 21.77–23.81 under SFK60, 2.26–6.52 under FGK0, 8.59–12.07 under FGK30, 12.93–16.15 under FGK45, and 17.81–19.46 μmol·mol-^−1^ under FGK60 (Fig. 1d).

Comprehensive analysis showed that Pn, Gs, Tr increased and Ci decreased significantly after the treatment of SF under the same potassium supply. Under the same straw return method, Pn, Gs and Tr values increased significantly with the potassium fertilization levels, while Ci decreased. The effects of straw return and potassium fertilizer on maize photosynthesis increased gradually from year to year.

### Effects of straw return and potassium fertilizer on dry matter of maize

**Figure 2:**
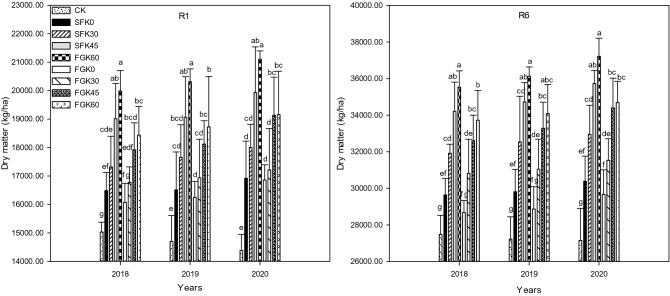
Effects of straw return methods and potassium fertilization levels on maize dry matter. Values followed by different letters in the same year indicated indicate statistical significance at α = 0.05 under different treatments. The same below.

We can see from Fig. 2, the straw return methods and potassium fertilization levels significantly increased (*p* ≤ 0.05) the maize dry matter accumulation. Compared with CK, under the treatments of SFK0, SFK30, SFK45, SFK60, FGK0, FGK30, FGK45 and FGK60, the dry matter of R1 and R6 stage increased by 1454.45, 2288.75, 3982.85, 4961.45, 1042.96, 1744.54, 2890.65, 3408.39 and 2152.43, 4433.55, 6726.72, 8051.51, 1195.76, 3337.79, 5121.77, 6247.56 kg/ha in 2018; the dry matter increased by 1812.69, 2959.44, 4370.19, 5615.94, 1545.06, 2238.06, 3421.11, 4028.64 and 2588.52, 5319.60, 7500.74, 8912.64, 1649.67, 3832.46, 6065.90, 6864.33 kg/ha in 2019; the dry matter increased by 2535.39, 3612.35, 5544.00, 6720.12, 2474.18,2827.94, 4749.86, 4769.66 and 3235.18, 5798.75, 8577.48, 10,071.83, 2515.75, 4386.39, 7256.61, 7536.91 kg/ha in 2020.

In short, under the same straw return method, the increase of maize dry matter from R1 to R6 improved significantly with the potassium level, potassium fertilizer could improve the maize dry matter accumulation ability. The maize dry matter of R1 to R6 increased significantly after the treatment of SF compared to FG under the same potassium supply. The promotion effect of straw return and potassium fertilizer on maize dry matter increased from year to year.

### Effects of straw return and potassium fertilizer on maize yield

**Figure 3:**
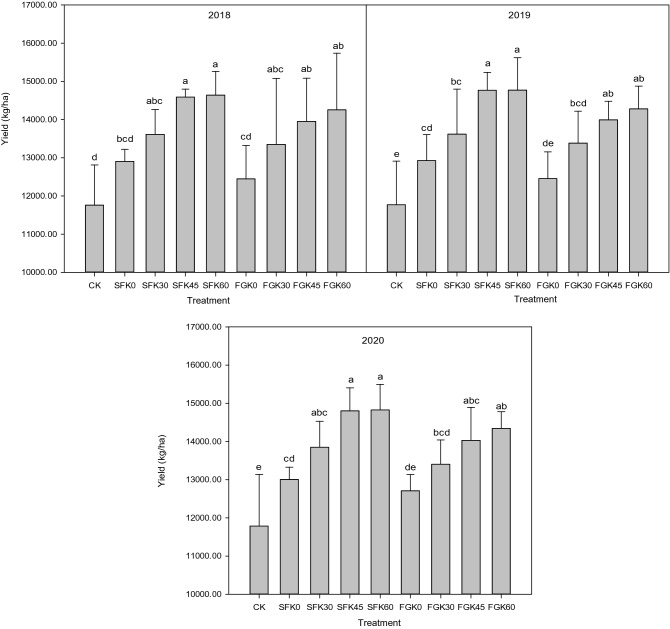
Effects of straw return methods and potassium fertilization levels on maize yield.

The straw return methods and potassium fertilization levels significantly influenced (*p* ≤ 0.05) the maize yield compared to CK, resulting in maize yield values that were higher than those of CK. Straw return and potassium supply increased maize yield. From 2018 to 2020, compared with CK, maize yield increased by 9.73–10.32% under SFK0, 15.68–17.47% under SFK30, 24.02–25.58% under SFK45, 24.46–25.76% under SFK60, 5.79–7.83% under FGK0, 13.51–13.72% under FGK30, 18.64–19.01% under FGK45, and 21.19–21.69% under FGK60 (Fig. 3).

The maize yield among treatments was as follows: SFK60 > SFK45 > FGK60 > FGK45 > SFK30 > FGK30 > SFK0 > FGK0 > CK. Compared to FG, the effect of SF on maize yield was more obvious. The maize yield increased significantly with the potassium fertilization levels under the potassium fertilization levels of 0–60 kg/ha in this test. The treatment of SFK60 recorded the highest average yield in the three-year test, which was 14,744.39 kg/ha. The maize yield in different planting years showed as follows: 2020 > 2019 > 2018, which indicated that the promotion effect of straw return and potassium fertilizer on maize yield increased from year to year.

### Correlation analysis of photosynthesis, dry matter accumulation and yield of maize

Pn, Gs, Tr and Ci were significantly correlated with dry matter accumulation. Pn, Gs and Tr were positively correlated with dry matter, while Ci was negatively correlated with the dry matter (Table 4). The results showed that the increase of Pn, Gs, Tr and the decrease of Ci could significantly improve maize dry matter. Dry matter was positively correlated with maize yield, indicating that the increase of dry matter accumulation could significantly improve maize yield. The increase of Pn, Gs, Tr and dry matter accumulation, as well as the decrease of Ci, could significantly increase maize yield.

**Table 4:** Correlation analysis of photosynthesis, dry matter accumulation and yield of maize under two straw return methods.

Method	Index	Pn (μmol·m^-2^·s^-1^)	Gs (mmol·m^-2^·s^-1^)	Tr (mmol·m^-2^·s^-1^)	Ci (μmol·mol^-1^)	Dry matter in R1(kg/ha)	Dry matter in R6 (kg/ha)	Yield (kg/ha)
SF	Pn (μmol·m^-2^·s^-1^)	1						
	Gs (mmol·m^-2^·s^-1^)	0.900**	1					
	Tr (mmol·m^-2^·s^-1^)	0.939**	0.982**	1				
	Ci (μmol·mol^-1^)	–0.933**	-0.995**	–0.989**	1			
	Dry matter in R1 (kg/ha)	0.965**	0.971**	0.979**	–0.981**	1		
	Dry matter in R6 (kg/ha)	0.945**	0.980**	0.992**	–0.986**	0.989**	1	
	Yield (kg/ha)	0.862**	0.988**	0.962**	–0.978**	0.948**	0.971**	1
FG	Pn (μmol·m^-2^·s^-1^)	1						
	Gs (mmol·m^-2^·s^-1^)	0.873**	1					
	Tr (mmol·m^-2^·s^-1^)	0.923**	0.978**	1				
	Ci (μmol·mol^-1^)	–0.912**	–0.970**	–0.986**	1			
	Dry matter in R1 (kg/ha)	0.881**	0.971**	0.947**	–0.948**	1		
	Dry matter in R6 (kg/ha)	0.887**	0.989**	0.984**	–0.978**	0.981**	1	
	Yield (kg/ha)	0.838**	0.975**	0.970**	–0.973**	0.930**	0.979**	1

Under the method of SF, the correlation coefficients of Pn, Gs, Tr, dry matter at R1 stage, dry matter at R6 stage and Ci with yield were 0.862, 0.988, 0.962, 0.948, 0.971 and −0.978; the correlation coefficients were 0.838,0.975,0.970,0.930,0.979 and −0.973 under the method of FG. The results showed that, under the method of SF, the correlation coefficients between dry matter of R1 stage, Pn, Gs, Ci with yield were higher than that under the method of FG, which indicated that SF could promote the correlation between the dry matter of R1 stage, Pn, Gs, Ci with yield. Under the method of FG, the correlation coefficients between the dry matters of R6 stage, Tr with yield were higher than that under the method of SF, which indicated that FG could promote the correlation between the dry matter of R6 stage, Tr with yield.

### Effects of straw return and potassium fertilizer on the profit of maize planting

Gross income is an important economic index that determines the profit or benefit that a farmer can obtain. On the other hand, net return reflects the actual income of the farmer. According to the average selling price of maize (1 yuan/kg) from 2018 to 2020, the net income of maize planting of different treatments was as follows: SFK45 > SFK60 > FGK60 > FGK45 > SFK30 > FGK30 > SFK0 > FGK0 > CK (Table 5). Compared to CK. the average net profit of maize planting in the three-year test increased by 421.26, 1049.07, 2014.82, 1980.44, 313.58, 1035.34, 1587.44, 1828.69 yuan/ha between the treatments of SFK0, SFK30, SFK45, SFK60, FGK0, FGK30, FGK45 and FGK60. Straw return and potassium supply increased the net profit of maize planting. The net profit of maize planting increased significantly after SF compared to FG under the same potassium supply. The treatment of SFK45 reached the maximum profit of maize planting, which was 2014.82 yuan/ha.

**Table 5:** Effects of straw return methods and potassium fertilization levels on the profit of maize planting.

Treatment	Expenditure (yuan/ha)			Total expenditure (yuan/ha)	Yield (kg/ha)	Gross income (yuan/ha)	Net profit (yuan/ha)
	Straw returning	Potash fertilizer	Other				
CK	0	0	1700	1700	11,773.95	11,773.95	10,073.95
SFK0	750	0	1700	2450	12,945.21	12,945.21	10,495.21
SFK30	750	120	1700	2570	13,693.02	13,693.02	11,123.02
SFK45	750	180	1700	2630	14,718.77	14,718.77	12,088.77
SFK60	750	240	1700	2690	14,744.39	14,744.39	12,054.39
FGK0	450	0	1700	2150	12,537.53	12,537.53	10,387.53
FGK30	450	120	1700	2270	13,379.29	13,379.29	11,109.29
FGK45	450	180	1700	2330	13,991.39	13,991.39	11,661.39
FGK60	450	240	1700	2390	14,292.64	14,292.64	11,902.64

## Discussion

Photosynthesis is the physiological basis for crop growth and yield formation^36^, which was mainly controlled by the cultivation measures and fertilizer^37,38^. Improving leaf physiological activity and photosynthetic efficiency can obtain higher dry matter and yield^39–41^. Studies have found that straw return can improve the photosynthetic capacity of maize^42,43^. Xia et al.^44^ showed that potassium fertilizer can promote the maize photosynthetic characteristics and achieve the purpose of improving maize yield by the increase of Pn, Gs, Tr and the decrease of Ci. In this study, compared with CK, both SF and FG could enhance photosynthesis, and SF had a better effect than FG. The improving effect of photosynthesis improved significantly with the increase of potassium fertilization levels. Straw returning and potassium fertilizer can significantly increase Pn, Gs and Tr, and decrease Ci. Pn, Gs and Tr increased most under SFK60 treatment, which were 7.31–11.44 μmol·m^−2^·s^−1^, 0.20–0.22 mmol·m^−2^·s^−1^ and 1.74–1.99 mmol·m^−2^·s^−1^. Ci decreased most under SFK60 treatment, which was 21.77–23.81 μmol·mol^−1^.

Dry matter accumulation is the key to yield formation of maize^45^. Studies have shown that straw return promoted dry matter accumulation of maize, and different straw return methods had different effects on dry matter and yield^46–48^. Potassium is one of the essential nutrients for maize growth, which plays an important role in promoting the accumulation of dry matter^49,50^. Han^51^ found that there was a significant positive correlation between maize dry matter and yield. In a certain range of potassium fertilizer application, dry matter accumulation and yield of maize improved with the increase of potassium fertilizer application. Compared with CK, both SF and FG could increase dry matter, and SF had a better effect than FG. The improving effect of dry matter improved significantly with the increase of potassium fertilization levels. Dry matter accumulation increased most under SFK60 treatment, which was 4961.45–10,071.83 kg/ha.

Studies have shown that both straw return and potassium fertilizer can increase the yield and income of maize^52,53^. In this paper, compared with CK, both SF and FG could increase the yield and income of maize, and SF had a better effect than FG. Maize yield increased most under SFK60 treatment, which was 24.46–25.76%. The Net profit of maize was the largest under SFK45 treatment, which was 12,088.77yuan/ha ($1868.92 per ha).

## Conclusion

In conclusion, the maize photosynthesis, dry matter accumulation, yield and net profit of maize planting were significantly increased by straw return and potassium supply. The promotion effect of straw return and potassium fertilizer on the above indexes increased from year to year. In this experiment, SFK60 was the most effective treatment to improve maize photosynthesis, dry matter accumulation and yield. Photosynthesis, dry matter and yield of SFK45 treatment were only a little smaller than SFK60. The treatment of SFK45 could maximize farmers' net profit from planting maize. The net profit could reach 12,088.77 yuan/ha, which was equivalent to $1868.92 per ha. Therefore, SFK45 was an effective way to ensure the stable and higher yields of maize and to maximize the income of farmers.
